# From stress to screen: relationship between negative life events and short video addiction among college students: a chain-mediated effect of depression and experiential avoidance

**DOI:** 10.3389/fpsyg.2025.1677941

**Published:** 2025-12-11

**Authors:** Yan Liu, Yangyang Zhan, Yaorong Liu

**Affiliations:** 1 School of Media and Communication, Shenzhen University, Shenzhen, China; 2 School of Mathematical Sciences, Shenzhen University, Shenzhen, China; 3 School of Digital Communication, Guangzhou Huashang College, Guangzhou, China

**Keywords:** short video addiction, negative life events, stressful events, depression, experiential avoidance

## Abstract

**Introduction:**

In contemporary society, short video addiction has become an increasingly prevalent behavioral disorder among young individuals, raising concerns about its underlying causes and development.

**Method:**

A survey was conducted on a sample of Chinese college students (*N* = 843) to explore the impact of negative life events on short video addiction, as well as the underlying mechanisms.

**Results:**

(1) Negative life events exert a significant positive influence on short video addiction among young people; (2) both depression and experiential avoidance serve as mediators in the relationship between negative life events and short video addiction; and (3) beyond their independent mediating effects, depression and experiential avoidance collectively function as a chained mediating mechanism in this process.

**Discussion:**

By elucidating the dynamic interplay between external environmental stressors and internal psychological processes, this research contributes to the theoretical under-standing of short video addiction among college students. Furthermore, it provides a valuable foundation for the development of targeted intervention strategies and mental health support programs aimed at mitigating the adverse effects of negative life events on digital behavior.

## 1 Introduction

In the digital age, short videos have become a dominant form of communication and entertainment, profoundly influencing social media and digital culture (Nguyen and Veer, 2024). According to an authoritative survey, in China, individuals spend an average of 168 min (over 2.5 h) per day watching short videos (Chen et al., 2024), with college students being the primary user demographic (Ye et al., 2024a; Ye et al., 2025). Short Video Addiction (SVA) is a psychological and behavioral condition resulting from frequent and excessive use of short video applications (Ye et al., 2023a; Li et al., 2024). It is characterized by a persistent or episodic preoccupation that leads to a strong dependence on short video content (Andreassen et al., 2016). Previous research has linked short video addiction to both physical and psychological health consequences (Chen et al., 2024; Qu et al., 2024). It can also distort an individual’s perception of time, potentially contributing to academic decline (Zahra et al., 2022). Given its widespread prevalence and adverse effects, researchers have investigated this issue from multiple perspectives. However, a review of the existing literature on short videos reveals several important limitations:

First, in contrast to the extensive research on traditional smartphone or social media addiction, studies focusing specifically on short video platforms are still relatively limited (Li et al., 2024; Qu et al., 2024; Xue et al., 2025). Further empirical investigation is required to enrich this field (Ye et al., 2024a; Ye et al., 2025; Li et al., 2025). Short videos have several unique characteristics. Key features include rapid dopamine-inducing stimuli, frequent engagement through fragmented time use, and intelligent algorithms that reinforce addiction (Nguyen and Veer, 2024). These features suggest that traditional addiction models and intervention strategies may not be fully applicable to short video addiction. Therefore, it is imperative to refine and innovate existing frameworks to enable more precise and targeted research.

Second, existing studies primarily focus on single factors—such as personal traits, social exclusion, or parent–child relationships—in relation to short video usage (Xue et al., 2025). However, little research has explored how multiple negative factors interact and jointly shape behavior. According to Individual-Environment Interaction theory, individual behavior stems from the interaction between external environmental factors and personal characteristics (Lerner et al., 2006). This implies that short-video addiction, as a maladaptive behavior, is likely driven by the cumulative effects of multiple external stressors and individual susceptibility. Ozaki et al. (2020) also believed that internal and external environments both have important influences on college students. Thus, when researchers choose research concepts and theoretical frameworks, they should not limit students’ experiences to the university campus. The results of the interaction between people and the environment are called proximal processes. Meanwhile, drawing on the macro perspective of the Psychology of Harmony and Harmonization (Di Fabio and Tsuda, 2018), Duradoni et al. (2022) proposed the Digital Life Balance (DLB) framework, which provides a more powerful lens for understanding this phenomenon. According to the DLB framework, technology use becomes problematic not only because of the amount of time spent online, but also because digital engagement disrupts the balance between one’s online and offline lives. In essence, dysfunctional internet use can be viewed as a disharmonious process between one’s online and offline lives (Duradoni et al., 2024a., 2024b). Research based on this framework shows that when individuals are unable to satisfy psychological needs such as mattering and control in their offline lives, they tend to become frustrated, which triggers problematic online behaviors (Soysal et al., 2024; Duradoni et al., 2024a; Duradoni et al., 2025; Tosti et al., 2025). Conversely, when these needs are met offline, the likelihood of dysfunctional internet use decreases (Lima-Costa et al., 2024; Aldbyani et al., 2025; Malas et al., 2025). Thus, understanding short video addiction may require examining individuals’ psychological conditions and the environments in which they are embedded.

Third, college students are a high-risk group for short video addiction (Li et al., 2024; Ye et al., 2025). Data show that the overall prevalence of internet addiction among Chinese college students is about 11%, which is significantly higher than rates reported in other countries (Shao et al., 2018). This is an alarming situation. On the one hand, college students tend to have flexible schedules and ample free time, providing conditions for frequent exposure to short videos (Qin et al., 2022; Xue et al., 2025). At the same time, their cognitive control systems are still developing, leaving them with weaker self-regulation and making them susceptible to algorithmically curated, attention-grabbing content (Yang, 2023). College students, at a critical stage of personal development and social integration, are particularly vulnerable to external environmental and societal changes (Zhang et al., 2024). In the current Chinese context, structural challenges persist, including an extended economic downturn, a shrinking job market, income inequality, and intensified social competition. These external pressures have deeply permeated the everyday lives of college students, manifesting as employment anxiety, family financial strain, and academic setbacks. Such negative life events may trigger psychological stress responses and behavioral deviations, including addictive behaviors. A recent exploratory study suggests that negative life events may directly contribute to short video addiction among college students (Pu et al., 2023; Xue et al., 2025). However, the mechanisms underlying this association remain unclear, and further empirical validation is needed to determine the strength and pathways of this influence.

Based on this, the present study draws on the Individual-Environment Interaction theory and the Digital Life Balance (DLB) framework to examine the combined effects of multiple variables within the current socioeconomic context, including external environmental risks (e.g., negative life events), individual psychological states (e.g., depression), and emotional regulation mechanisms (e.g., experiential avoidance). This study aims to reveal the underlying mechanisms of short video addiction among college students, provide a more comprehensive theoretical perspective, and generate empirical evidence for effective prevention and intervention strategies.

## 2 Literature review and research hypothesis

### 2.1 Negative life events and short video addiction

Negative life events refer to stressful and unpleasant experiences that create pressure and tension in an individual’s life (Garnefski et al., 2001). For college students, these events typically include academic stress, interpersonal relationship problems, and family crises. As a negative environmental factor, negative life events can have a significant impact on an individual’s psychological well-being and behavior (Updegraff and Taylor, 2021). Previous studies have shown that the occurrence of negative events, such as interpersonal relationship problems, emotional difficulties, or learning obstacles, can predict internet addiction behaviors among college students (Kardefelt-Winther, 2014; Xue et al., 2025). Prievara et al. (2019) also noted that societal-level factors play a critical role in determining the dysfunctional use of emerging technologies.

In addition, from the perspective of the psychology of harmony, harmony refers to the process by which an individual integrates the body, mind, and spirit, as well as diverse life goals, into a coherent, well-functioning whole (Di Fabio and Tsuda, 2018). Inspired by this theory, Duradoni et al. (2022) proposed the concept of Digital Life Balance (DLB), emphasizing that maintaining a harmonious relationship between digital and real-life experiences is key to healthy digital use. The theory suggests that individuals experiencing uncertainty, helplessness, or reduced autonomy in their offline lives tend to seek compensatory experiences in digital contexts that provide a sense of control and achievement. Over time, however, this coping strategy can evolve into maladaptive or problematic behavior, leading to psychological imbalance and excessive digital media use (Duradoni et al., 2024a). In short, an imbalance between one’s online and offline lives can lead to the dysfunctional use of information and communication technologies (ICTs) (Soysal et al., 2024; Tosti et al., 2025). Recent findings also indicated that the frustration of social and control needs significantly increases the risk of dysfunctional internet use (Lima-Costa et al., 2024; Aldbyani et al., 2025; Malas et al., 2025). In such contexts, digital platforms become central arenas for satisfying these needs, which can lead to addiction (Aldbyani et al., 2025; Duradoni et al., 2025). This suggests that negative life events, as sources of stress, may undermine college students’ fulfillment of social needs and sense of self-efficacy in the real world, weaken their psychological balance, and consequently heighten their reliance on digital platforms. Therefore, this study hypothesizes that:

*H1*: Negative life events can positively predict short video addiction among college students.

### 2.2 Mediating role of depression

Negative life events are objectively stressful circumstances that can also lead to psychological distress. Extensive empirical evidence shows that negative life events often trigger emotions such as depression, loneliness, and helplessness (Zhou et al., 2025). According to the Individual-Environment Interaction theory, individual behavior stems from the interaction between adverse external conditions and internal vulnerabilities. In other words, the result of the interaction between the individual and the environment leads to one’s development. When external stressors exceed an individual’s coping capacity, psychological strain and behavioral dysregulation are likely to occur (Lerner et al., 2006; Bronfenbrenner, 2013). Therefore, negative life events may contribute to short video addiction among college students through psychological mechanisms such as depression.

On the one hand, depression, defined as a state of low mood, distress, or sadness, is recognized as a significant risk factor for mental and physical health in adolescents (Holden, 2000). Research indicates that adolescents are more likely to experience elevated levels of depression when facing challenges such as academic pressure, family conflicts, or social isolation (Gollust et al., 2008). Geng’s research suggests that as young people experience an increasing number of negative life events, they become more vulnerable to developing negative emotions, including anxiety and depression (Geng et al., 2020).

On the other hand, depression forms the foundation for addictive disorders (Qu et al., 2024). Depression has been shown to be significantly and positively correlated with various addictive behaviors (such as Internet addiction, smartphone addiction, and social media addiction; Busch and McCarthy, 2021). Anxious individuals often resort to frequent internet use to alleviate worry and uncertainty, which subsequently leads to increasingly dysfunctional internet behaviors (Yao et al., 2023). Recent research also suggests that depression and anxiety may act as risk factors for short video addiction, as an emerging subset of problematic Internet use, among Chinese college students (Zhang et al., 2024; Li et al., 2025). For example, a study by Yao et al. (2023) indicates that depression can predict short-form video addiction 2 months later. Furthermore, Ye et al. (2024a) also points out that short video addiction stems from negative emotions in real life. Conversely, other studies suggest that short video addiction significantly increases an individual’s level of depression (Qu et al., 2024; Yu et al., 2024). Zhang and Bian (2021) examined the brain structures underlying the link between pathological internet use and anxiety using magnetic resonance imaging. In conclusion, the inconsistencies in existing findings underscore the need for further research into the relationship between depressive symptoms and short video addiction. Based on this, we hypothesize that:

*H2*: Depression mediates the relationship between negative life events and short video addiction among college students.

### 2.3 Mediating role of experiential avoidance

Experiential avoidance (EA) is a key concept in psychological research, originating from Acceptance and Commitment Therapy (ACT) (Gámez et al., 2011). It refers to an individual’s tendency to escape, suppress, or modify unpleasant emotions, memories, or thoughts, even when such efforts may lead to long-term negative consequences (Hayes, 2004). According to the Experiential Avoidance Model, external distressing events often evoke negative emotions, resulting in intense psychological discomfort (Chapman et al., 2006). This discomfort can be difficult to endure, leading individuals to attempt to escape these unpleasant psychological experiences (Kashdan et al., 2013). Thus, negative life events may significantly increase an individual’s tendency toward experiential avoidance. Related studies have also demonstrated a positive correlation between the frequency of negative life events and the tendency toward experiential avoidance (He et al., 2021). When confronted with external stressors, escapism can be a distraction and coping mechanism (Kuo et al., 2016).

Meanwhile, avoidance-oriented expectations have been identified as a proximal factor in the development of internet addiction. For example, He et al. (2021) have identified a significant positive correlation between experiential avoidance and problematic behaviors. Research by Wei et al. (2023) further highlights that experiential avoidance is a major contributor to non-substance addictions among youth. Previous research also indicates that detachment, characterized by social and emotional avoidance, is associated with higher levels of internet use (Laier et al., 2018). Individuals may turn to digital platforms to avoid negative self-perceptions or to escape unresolved painful feelings, such as abandonment, worthlessness, and emptiness (Ye et al., 2024b; Duradoni et al., 2025). This, in turn, increases their dependence on these platforms (Aldbyani et al., 2025; Malas et al., 2025). Based on this, the following hypothesis is proposed:

*H3*: Negative life events influence short video addiction through the mediating effect of experiential avoidance.

### 2.4 Effects of depression on experiential avoidance

Experiential avoidance allows individuals to temporarily escape unwanted internal experiences, making it a common strategy for alleviating negative emotions (Sung et al., 2020). A substantial body of research has found a strong association between depression and experiential avoidance (He et al., 2021; Wei et al., 2023). Furthermore, depression may indirectly contribute to short video addiction through the mediating role of experiential avoidance.

According to General Strain Theory (GST), strained interpersonal relationships or stressful events can trigger negative emotions such as depression and anxiety (Agnew, 1992). In response, individuals activate self-defense mechanisms and adopt various coping strategies to mitigate the effects of negative life events. The DLB framework also suggests that digital media are often used as a means of coping with offline life’s challenges (Soysal et al., 2024; Duradoni et al., 2025). For college students who struggle to effectively manage negative life events, this digital “emotional refuge” becomes even more appealing (Nong et al., 2023; Aldbyani et al., 2025; Tosti et al., 2025). For example, short videos can serve as a form of temporary emotional relief (Ye et al., 2022; Ye et al., 2023a). People often alleviate feelings of depression by watching short, entertaining videos. Through immersive engagement—such as liking, commenting, and participating in live streams—they temporarily disconnect from real life and escape feelings of helplessness and loneliness (Ye et al., 2023b; Nguyen and Veer, 2024). The instant gratification associated with these behaviors may further increase their reliance on short videos, potentially leading to addiction. A recent study by Ye et al. (2025) also suggests that short videos provide users with high emotional value. The stimulating content can trigger the release of dopamine in the brain, producing what is known as “TikTok brain,” which compels users to repeatedly seek out this sensation. However, empirical research on the direct relationships between negative life events, depression, experiential avoidance, and short video addiction remains limited. Therefore, the following hypothesis is proposed:

*H4*: Negative life events influence short video addiction through the sequential mediating effects of depression and experiential avoidance.

### 2.5 The present study

**Figure 1:**
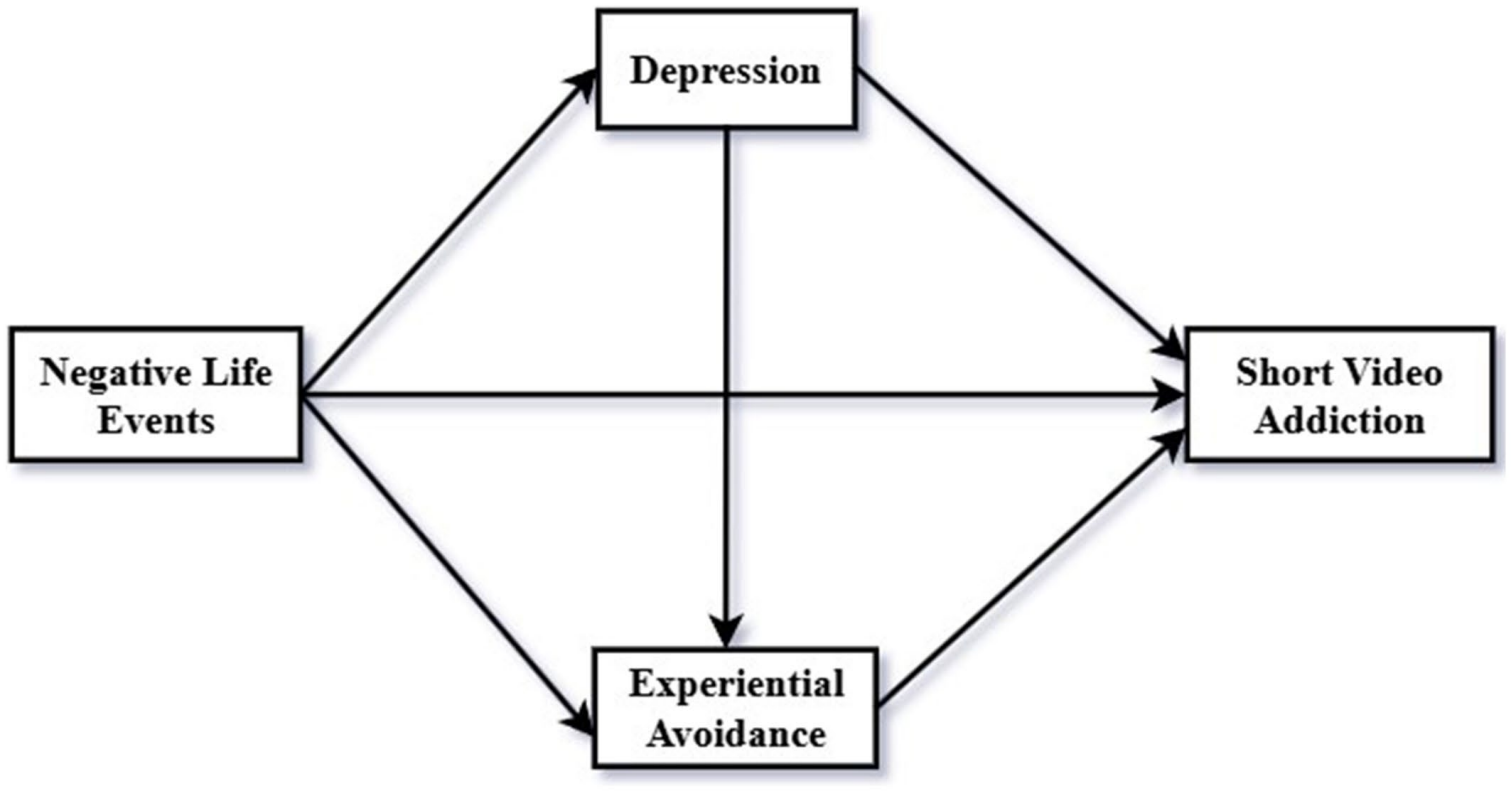
The proposed model of the current study.

This study considers both external factors and individual psychological factors, using the Individual-Environment Interaction theory and the Digital Life Balance (DLB) framework. Specifically, the study addresses the following key questions: (a) Do negative life events positively predict short video addiction? (b) Does depression mediate the relationship between negative life events and short video addiction? (c) Does experiential avoidance mediate the relationship between negative life events and short video addiction? (d) Do depression and experiential avoidance jointly mediate the relationship between negative life events and short video addiction through a chain-mediated effect? The proposed multiple mediation model is illustrated in Figure 1.

## 3 Materials and methods

### 3.1 Participants and procedures

We used the snowball sampling technique to distribute the questionnaires via the Wenjuanxing platform (www.wjx.cn). In China, Wenjuanxing is widely used by researchers investigating media usage behavior among youth groups (Zhang et al., 2025; Ye et al., 2025). Its key advantages—high compatibility, real-time monitoring, and robust privacy protection—provide essential technical support for efficient research.

To enhance the diversity and representativeness of the sample, we adopted a multi-channel, multi-platform distribution strategy. The questionnaire link and QR code were distributed through major social media platforms, including Weibo, WeChat, QQ groups, and Xiaohongshu communities. This approach integrated convenience sampling with the characteristics of the target population to ensure broad coverage of students of different ages, genders, regions, and social backgrounds. In addition, drawing on insights from previous research (Yu et al., 2024), we included a description of short video usage at the beginning of the questionnaire. This definition clarified that short video use encompasses activities such as subscribing, commenting, and creating videos on short video applications (e.g., Douyin, Kuaishou, Bilibili, Xigua Video) and social media platforms with video-sharing capabilities (e.g., QQ, WeChat, Xiaohongshu). We established the following screening criteria to ensure data quality and questionnaire validity: (1) multiple submissions from the same IP address, (2) uniform responses across all questions, (3) a completion time of less than 120 s, and (4) failure to pass attention checks (e.g., “Please select ‘Strongly Disagree’”).

The survey was entirely anonymous and voluntary, and participants could withdraw at any time. Upon completion of the questionnaire, participants were randomly awarded a cash incentive ranging from 3 to 5 RMB.

The study was conducted over 2 months, from October 11, 2024, to December 10, 2024. A total of 900 Chinese college students were recruited for the survey, resulting in 843 valid responses. The sample consisted of 369 males (43.8%) and 474 females (56.2%) aged between 18 and 23 years (M = 20.29, SD = 1.70). It is generally accepted that the ratio of subjects to number of items should not be less than 5:1, with 10:1 being more appropriate (Everitt, 1975; Gorsuch, 2014). In addition, prior to participant recruitment, we conducted a power analysis using G*Power 3.1 (Faul et al., 2009), which indicated that at least 460 participants were required to achieve adequate statistical power. Therefore, the sample size in this study sufficiently meets the required standard, ensuring the reliability of the research results.

### 3.2 Measures

#### 3.2.1 Negative life events

Negative life events were measured using the Adolescent Stressful Life Events Scale, revised by Li et al. (2010), which assesses the occurrence and severity of negative life events over the past year. The scale consists of 16 items covering areas such as family financial difficulties, parental divorce, interpersonal relationships, and academic performance. Responses were recorded on a 6-point Likert scale, where 0 indicates that the event did not occur, and scores from 1 to 5 reflect the perceived impact of the event (0 = “no impact” to 5 = “extremely severe”). Higher scores indicate a greater perceived impact of negative life events. The scale has been widely used in trauma-related surveys among general child and adolescent populations in China (Zhou et al., 2025), demonstrating strong reliability and validity. In this study, the Cronbach’s *α* of the scale was 0.89. The results indicated satisfactory structural validity: *χ*^2^/df = 2.808, RMSEA = 0.046, RMR = 0.045, GFI = 0.958, AGFI = 0.945, CFI = 0.940.

#### 3.2.2 Depression

Depression was measured using the Depression subscale of the Chinese version of the DASS-21, revised by Gong et al. (2010). This subscale consists of 7 items, including representative statements such as “I could not feel enthusiastic about anything,” “I felt depressed and blue,” and “I felt that life was meaningless.” Responses were recorded on a 4-point Likert scale ranging from 0 (“does not apply at all”) to 3 (“applies very much”). Higher total scores indicate greater levels of depression. The scale is widely used to assess and differentiate clinical and non-clinical emotional disorders and has demonstrated strong reliability and validity (He et al., 2021; Wei et al., 2023). In this study, the Cronbach’s *α* of the scale was 0.82. The results indicated satisfactory structural validity: *χ*^2^/df = 2.900, RMSEA = 0.047, RMR = 0.019, GFI = 0.986, AGFI = 0.972, CFI = 0.984.

#### 3.2.3 Acceptance and action questionnaire

Experiential avoidance was measured by the Acceptance and Action Questionnaire-II (AAQ-II), developed by Bond et al. (2011) and translated and revised into Chinese by Cao et al. (2013). The scale evaluates the extent to which individuals avoid internal private experiences they are unwilling to confront. It consists of 7 items, including representative statements such as “My painful memories prevent me from living a full life,” “Emotions interfere with my life,” and “Certain feelings frighten me.” Responses were rated on a 7-point Likert scale ranging from 1 (“strongly disagree”) to 7 (“strongly agree”), with higher total scores indicating greater levels of avoidance. Previous studies have demonstrated that the Chinese version of the AAQ-II exhibits strong reliability and validity (He et al., 2021). In this study, the Cronbach’s α of the scale was 0.89. The results indicated satisfactory structural validity: *χ*^2^/df = 1.553, RMSEA = 0.026, RMR = 0.031, GFI = 0.993, AGFI = 0.985, CFI = 0.997.

#### 3.2.4 Short video addiction scale

Short video addiction was measured using the Bergen Short-form Video Addiction Scale. This scale was adapted from the Bergen Social Media Addiction Scale (Andreassen et al., 2016), and the word “social media” was replaced by “short video.” To date, this scale has been applied to diverse populations, including middle and high school students, college students, and older adults, and has consistently demonstrated strong reliability, validity, and applicability (Qin et al., 2022; Qu et al., 2024). The instrument consists of 6 items rated on a 5-point Likert scale (1 = “very rarely,” 5 = “very often”). Example items include “I lose sleep over spending more time on this short video app,” “I have difficulties focusing on my studies due to this short video app,” and “I feel anxious if I cannot access this short video app.” Higher total scores indicate greater addictive short video use. In this study, the Cronbach’s α of the scale was 0.87. The results indicated satisfactory structural validity: *χ*^2^/df = 1.654, RMSEA = 0.028, RMR = 0.012, GFI = 0.994, AGFI = 0.987, CFI = 0.997.

### 3.3 Analytical strategy

Data were processed using SPSS 26.0 and PROCESS 3.5. First, descriptive statistics and Pearson correlations were conducted to examine the relationships between negative life events, depression, experiential avoidance, and Short video addiction. Second, we used PROCESS version 3.5, an SPSS macro developed by Hayes (2017), to test the research hypotheses. Given that the hypotheses proposed a serial mediation model with two mediators, Model 6 from the PROCESS templates was selected for statistical analysis. Gender and age were included as covariates. All regression coefficients were tested using the bias-corrected percentile Bootstrap method, and the 95% confidence interval (CI) was calculated through 5,000 resampled iterations.

## 4 Results

### 4.1 Common method bias and data normality

Self-reported data may introduce common method bias. To enhance the scientific rigor of this study, Harman’s single-factor test was performed to assess this potential bias. The analysis identified seven factors with eigenvalues greater than 1. The first factor accounted for only 25.99% of the total variance, well below the critical threshold of 40% (Hair et al., 2010). These results suggest that general method bias was not a significant problem in this study.

The variables followed a normal distribution as their skewness and kurtosis values were within acceptable ranges (skewness < |2.0| and kurtosis < |7.0|) (Byrne, 2001). In addition, the Variance Inflation Factor (VIF) values for all variables were below the critical threshold of 5 (Hair et al., 2010; Byrne, 2001), indicating no collinearity in the model. These results confirmed that the data were suitable for further analysis.

### 4.2 Correlation analysis

The results of the descriptive analysis and correlation tests are presented in Table 1. Significant correlations were found between negative life events and depression (*r* = 0.37, *p* < 0.01), experiential avoidance (*r* = 0.36, *p* < 0.01), and short video addiction (*r* = 0.38, *p* < 0.01). In addition, depression was positively correlated with both experiential avoidance (*r* = 0.32, *p* < 0.01) and short video addiction (*r* = 0.43, *p* < 0.01). Furthermore, experiential avoidance was found to be significantly related to short video addiction (*r* = 0.36, *p* < 0.001). There are no significant differences based on gender. These significant relationships among the primary variables provide empirical support for the proposed sequential mediation model.

**Table 1:** Correlation analysis among all variables.

Variables	M	SD	1	2	3	4
1. Negative life events	3.10	0.70	1			
2. Depression	1.14	0.62	0.37^**^	1		
3. Experiential avoidance	4.27	1.15	0.36^**^	0.32^**^	1	
4. Short video addiction	2.96	0.75	0.38^**^	0.43^**^	0.36^**^	1

**p < 0.01.

### 4.3 Chain mediation model test

The results of the regression analysis (see Table 2) indicate that negative life events significantly positively predicted depression (*β* = 0.34, *p* < 0.001) and experiential avoidance (β = 0.47, *p* < 0.001). Depression, in turn, significantly positively predicted experiential avoidance (β = 0.38, *p* < 0.001). When negative life events, depression, and experiential avoidance were simultaneously included in the regression model, negative life events had a significant direct effect on short video addiction (β = 0.21, *p* < 0.001). In addition, depression positively predicted short video addiction (β = 0.36, *p* < 0.001), and experiential avoidance also positively predicted short video addiction (β = 0.13, *p* < 0.001). Hypotheses 1–3 are confirmed.

**Table 2:** Testing the mediation model.

Regression equation		Fitness index			Significance of regression coefficients			
Outcome variables	Predictor variables	*R*	*R*^2^	*F*	*β*	SE	LLCI	ULCI
Depression		0.38	0.14	46.63				
	Gender				0.03	0.04	−0.05	0.11
	Age				0.03	0.02	−0.02	0.08
	Negative Life Events				0.34***	0.03	0.28	0.39
Experiential avoidance		0.41	0.17	42.94				
	Gender				0.02	0.07	−0.12	0.17
	Age				−0.01	0.04	−0.09	0.08
	Negative Life Events				0.47***	0.06	0.36	0.58
	Depression				0.38***	0.06	0.26	0.51
Short video addiction		0.52	0.27	62.16				
	Gender				−0.01	0.05	−0.01	0.08
	Age				0.01	0.03	−0.05	0.06
	Negative life events				0.21***	0.04	0.14	0.28
	Depression				0.36***	0.04	0.28	0.43
	Experiential avoidance				0.13***	0.02	0.08	0.17

***p < 0.001.

To determine the significance of the mediating effects, the Bootstrap method was used to test for multiple mediating effects, and the results revealed that the upper and lower limits of the 95% confidence intervals for each mediating path did not contain 0.

The mediation analysis (see Table 3 and Figure 1) revealed that depression and experiential avoidance mediated the relationship between negative life events and short video addiction. The mediation effect was 0.20, accounting for 48.78% of the total effect of negative life events on short video addiction (0.41). Specifically, the mediating effect was generated through three indirect pathways: First, via the pathway “Negative Life Events → Depression → Short Video Addiction,” with an effect value of 0.12 (Bootstrap 95% CI, [0.09, 0.15]). Second, via the pathway “Negative Life Events → Experiential Avoidance → Short Video Addiction,” with an effect value of 0.06 (Bootstrap 95% CI, [0.04, 0.08]). Third, via the pathway “Negative Life Events → Depression → Experiential Avoidance → Short Video Addiction,” with an effect value of 0.02 (Bootstrap 95% CI, [0.01, 0.03]).

**Table 3:** Pathway analysis.

Type of Paths	Pathways	Effect	BootSE	95% CI	
				BootLLCI	BootULCI
Indirect effect	NLE → DE → SVA	0.12	0.01	0.09	0.15
	NLE → EA → SVA	0.06	0.01	0.04	0.08
	NLE → DE → EA → SVA	0.02	0.004	0.01	0.03
Total indirect effect		0.20	0.02	0.15	0.24
Total effect		0.41	0.03	0.34	0.48

NLE, negative life events; DE, depression; EA, experiential avoidance; SVA, short video addiction.

These findings suggest that both depression and experiential avoidance play independent mediating roles and function as part of a chain mediation effect in the relationship between negative life events and short video addiction (see Table 3). Hypotheses 4 are confirmed.

## 5 Discussion

This study, situated in a specific social context, examines the complex mechanisms by which negative life events influence short video addiction and validates the critical role of depression and experiential avoidance in this process.

### 5.1 The direct effect of negative life events on short video addiction

This study identified a significant positive relationship between negative life events and short video addiction among college students, extending existing research into the area of short video addiction. There are two possible explanations for this relationship:

First, the culturally ingrained traits of modesty and introversion in Chinese society often lead college students to internalize stress rather than seek external support when facing negative life events, such as academic failure, career challenges, interpersonal conflicts, or family unemployment. Short video platforms offer a relatively anonymous and informal space for self-expression, serving as a crucial outlet for alleviating stress and fostering positive emotional experiences (Kardefelt-Winther, 2014; Zhang et al., 2025). By interacting with online streamers, students can share their feelings, seek emotional validation, and receive support, thereby easing their psychological burden. This finding is also consistent with the macro framework of the Psychology of Harmony (Di Fabio and Tsuda, 2018) and is particularly supported by the Digital Life Balance model. Specifically, compensating for unmet needs for control and sociability in the offline world can lead to disharmonization between online and offline life (Duradoni et al., 2024a; Duradoni et al., 2025; Aldbyani et al., 2025; Tosti et al., 2025; Malas et al., 2025). Similarly, Xue et al. (2025) also found that as the number of adverse experiences an individual has increases in daily life, the likelihood of mild, moderate, or severe short video addiction rises by 1.17 to 4.68 times.

Second, according to Media Dependency Theory, the more functions and services a medium provides, the stronger the audience’s dependency on it becomes (Ball-Rokeach and DeFleur, 1976). When individuals excessively rely on a particular medium to meet their emotional or social needs, this dependence can adversely affect their psychological well-being and behavior. This means that if college students become immersed in short video platforms and use them as their primary “comfort zone” for stress relief and emotional support, they are highly susceptible to developing a short video addiction. This is also consistent with recent studies. For example, Zhang et al. (2025) found that stress heightens medical students’ need for online entertainment and emotional compensation, leading to more frequent short-video use and ultimately resulting in addiction.

### 5.2 The mediating role of depression

This study found that depression mediated the relationship between negative life events and short video addiction among college students.

First, stressful life events are a well-established predictor of depressive symptoms (Zhou et al., 2025). Such negative experiences expose college students to adversity, exacerbating their psychological turmoil and emotional overload (Copeland et al., 2024). As these challenges intensify, negative emotions also become more pronounced. Individuals with poor emotional regulation are especially vulnerable to depression, further increasing their risk of mental health problems and behavioral disorders. This is consistent with Duradoni’s research, which shows that higher levels of disinhibition, negative affect, detachment, and psychoticism are associated with lower levels of Digital Life Balance (DLB) and higher levels of internet addiction. Qu et al. (2024) also demonstrated a significant relationship between depression and short video addiction.

Second, the findings are also supported by the Limited Self-Control Theory, which posits that self-control is a finite resource (Muraven et al., 1998). Once this resource is depleted, it leads to failures in self-regulation during subsequent tasks (Baumeister, 2002). In fact, many problematic behaviors can be attributed to a lack of self-control (González-Fuente and Moral-Jiménez, 2024; Li et al., 2025). This suggests that when college students encounter various stressful life events, their already limited self-control resources are rapidly exhausted as they attempt to manage negative emotions. This depletion further weakens their ability to regulate short video consumption. Impaired self-control makes it more difficult to manage impulses, increasing susceptibility to instant gratification. Consequently, students struggle to resist the allure of engaging content on short video platforms, heightening the risk of addiction. This finding aligns with Li et al. (2025), who found that negative emotional states can reduce self-control in a variety of ways (influencing persistence, focus, and feelings of exhaustion) and increase short video addiction tendencies. Similarly, Liao (2024) and Ye et al. (2025) highlighted that addicted users struggle to maintain their attention because they experience more attention control deficits when watching short videos.

### 5.3 The mediating role of experiential avoidance

The results also indicated that experiential avoidance mediated the relationship between negative life events and short video addiction among college students. This relationship can be explained from the following perspectives.

First, according to the Experiential Avoidance model, when individuals encounter painful, distressing, or threatening internal experiences, they often adopt various strategies to minimize or escape these unwanted states—even when such strategies may lead to negative long-term consequences (Chapman et al., 2006). This means that prolonged exposure to stressful environments often leads individuals to experience various negative emotions, including anger and depression. Under the combined influence of high emotional intensity, limited psychological resources, and restricted regulatory strategies, individuals may resort to short video consumption as a low-cost strategy for experiential avoidance. This is consistent with previous studies, which show that users increase their problematic online activity in an attempt to escape negative emotions arising from daily life and experience more positive ones (Li et al., 2025; Zhang et al., 2025). However, in the long term, such addictive behavior only exacerbates negative emotions rather than positive ones, ultimately reducing well-being and life satisfaction (Longstreet et al., 2019; Ye et al., 2024a).

Second, this conclusion is also strongly supported by the Stimulus–Organism–Response (S-O-R) framework. This theory posits that external environmental stimuli do not directly lead to behavioral outcomes. Rather, they must first be processed through an individual’s internal cognitive and emotional systems, which give rise to specific behavioral responses (Mehrabian and Russell, 1974). In this study, experiential avoidance functions as the key organism variable (O), illuminating how negative life events (the external stimulus, S) are internalized and ultimately transformed into the behavioral response (R) of short-video addiction. This suggests that short video platforms have largely become an outlet for college students to avoid real-world stress and emotional distress. This finding aligns with existing literature. Previous studies have also shown that avoiding emotional and social situations may be linked to heightened negative affect and frustrated social needs (Lima-Costa et al., 2024; Malas et al., 2025). This makes short-form videos a coping tool (Kardefelt-Winther, 2014; Hu and Huang, 2024), as it allows for limited interaction within a controlled environment and provides emotionally manageable settings (Malas et al., 2025; Akhtar et al., 2025). However, the need for gratification amplifies this behavior (Feng et al., 2025).

### 5.4 The chain mediating role of depression and experiential avoidance

This study found that depression and experiential avoidance function as sequential mediators in the relationship between negative life events and short video addiction among college students. The findings suggest that negative life events heighten the risk of short video addiction through the combined influence of emotional responses (depression) and coping mechanisms (experiential avoidance). Specifically, stressful life events evoke depressive emotions, which subsequently lead to an increased tendency toward experiential avoidance. In this context, short video platforms provide a readily accessible means of facilitating avoidance behaviors. However, this pattern can further reinforce individual’s path dependency, ultimately perpetuating a vicious cycle of “negative events → depression → avoidance → short video addiction.”

This mechanism is also supported by the Individual-Environment Interaction theory, which posits that individual behavior results from the interplay between external environmental conditions and personal characteristics (Lerner et al., 2006; Bronfenbrenner, 2013). In the proposed model, negative life events create adverse external pressures, whereas depression and experiential avoidance represent the internal psychological and emotional responses individuals adopt when coping with such conditions. Short video addiction emerges as a maladaptive outcome of the interaction between a “high-stress environment” and “low adaptive psychological functioning.”

Additionally, the Digital Life Balance (DLB) framework offers a deeper theoretical lens for understanding this chain of effects. The DLB framework emphasizes that harmony and need fulfillment in offline life are prerequisites for maintaining healthy digital behaviors (Duradoni et al., 2022; Tosti et al., 2025). When negative life events disrupt this balance and trigger psychological disequilibrium—such as depression—individuals tend to seek digital compensation as a way to restore inner order. In this process, experiential avoidance becomes a functional regulatory strategy, motivating individuals to use short-video platforms as an “alternative center” for escaping real-world distress and regaining a sense of control. Over time, this compensatory use becomes distorted, evolving from coping to addiction. This suggests that when external disruptions undermine the well-being of college students, those who lack effective offline coping strategies may turn to short-video apps to fulfill unmet psychological or social needs (Kuo et al., 2024; Ye et al., 2024a). However, this may intensify their psychological distress, creating a self-perpetuating cycle of dependence. Therefore, the findings of this study underscore the importance of identifying more adaptive emotional regulation strategies, especially in today’s context of uncertainty and intense social competition.

## 6 Implications

### 6.1 Theoretical implications

First, this study differs from previous research that predominantly focused on psychological vulnerability factors. Instead, it incorporates the specific social context and explores the intrinsic links between environmental risks, emotional reactions, coping mechanisms, and short video addiction among college students. Results show that negative life events significantly predict short video addiction. This finding makes a valuable contribution to the existing scholarly discourse on short video addiction by emphasizing the interaction between external environmental conditions and internal psychological processes in shaping this behavior. The study further substantiates the Individual-Environment Interaction Theory.

Second, the findings further enhance the explanatory power of the DLB model, demonstrating that maladaptive digital engagement can be understood as compensation for unmet needs—especially control and relatedness—in offline contexts. In other words, this study extends the application of the Digital Life Balance (DLB) framework to research on short-video addiction, offering novel perspectives and directions for future studies on addiction mechanisms and theoretical model development.

Third, this study further uncovers the critical role of experiential avoidance as a bridge in the relationship between negative life events and short video addiction. That is, the primary reason that negative life events lead to short video addiction is the use of passive coping strategies, specifically experiential avoidance. It deepens the scientific understanding of short video addiction and provides empirical support for interventions targeting digital addiction among college students. Such interventions should focus on cultivating diverse emotional regulation strategies and strengthening psychological coping skills.

### 6.2 Practical implications

On one hand, the study underscores the significant impact of negative life events on college students’ mental health and digital engagement dysregulation, suggesting that universities should prioritize fostering students’ psychological resilience in coping with adversity in mental health education. Concurrently, based on the Digital Life Balance (DLB) framework, universities should adopt a harmony-based intervention approach designed to restore a dynamic equilibrium between students’ online and offline lives. Specifically, universities can enhance the appeal of real-world interactions by organizing diverse extracurricular activities, sports competitions, and social practice programs. Such initiatives aim to help students to reduce their dependence on digital technologies and rebuild their sense of agency and relatedness in the physical world.

On the other hand, students should embrace negative experiences rather than avoid or suppress them. Given the current challenging job market and economic downturn, we encourage students to actively learn stress management techniques, such as deep breathing, mindfulness meditation, and physical exercise (Liu et al., 2023; Yosep et al., 2024). These practices not only help alleviate emotional fluctuations but also contribute to building psychological resilience (Cavour-Więcławek and Rogowska, 2024). In addition, participation in volunteer or community activities can enrich students’ lives and provide healthy outlets for negative emotions, thereby reducing over-reliance on short video platforms and other forms of entertainment.

## 7 Limitations and future direction

Although this study yielded valuable findings, it has certain limitations that should be addressed in future research: First, the study employed a cross-sectional design, which inherently limits the ability to draw causal inferences. Future research directions could be expanded to longitudinal analyses of digital life balance and verify the long-term efficacy of intervention strategies. Second, given that college students are a high-risk group for short video addiction, this study focused primarily on that population, resulting in a relatively homogeneous sample. Additionally, the use of snowball sampling limits the breadth and randomness of the sample. Future research could expand the sample to include participants from diverse ages and social backgrounds and employ probabilistic sampling techniques to enhance the generalizability of the findings. Finally, as all data in this study were self-reported, there may be unavoidable biases. Future research could consider leveraging experimental manipulation or neuroscience techniques (e.g., electroencephalography or functional magnetic resonance imaging) to explore the research topic from multiple perspectives.

## 8 Conclusion

The main findings of the study are as follows: (1) Negative life events have an overall positive effect on short video addiction among college students; (2) Negative life events positively influence short video addiction among college students through depression; (3) Negative life events positively influence short video addiction among college students through experiential avoidance; (4) Negative life events influence short video addiction among college students through the sequential mediating effects of depression and experiential avoidance.

## Data Availability

The original contributions presented in the study are included in the article/supplementary material, further inquiries can be directed to the corresponding author.

## References

[ref1] Agnew R. (1992). Foundation for a general strain theory of crime and delinquency. Criminology 30, 47–88.

[ref2] Andreassen C. S. Billieux J. Griffiths M. D. Kuss D. J. Demetrovics Z. Mazzoni E. . (2016). The relationship between addictive use of social media and video games and symptoms of psychiatric disorders: a large-scale cross-sectional study. Psychol. Addict. Behav. 30, 252–262. doi: 10.1037/adb0000160, 26999354

[ref3] Aldbyani A. Wang G. Chuanxia Z. Alhimaidi A. (2025). Dispositional mindfulness is associated with lower smartphone addiction through digital life balance among Chinese university students. Front. Psychol. 16:1653620. doi: 10.3389/fpsyg.2025.1653620, 41181686 PMC12571664

[ref4] Akhtar N. Islam T. Hameed Z. Ghaffar A. Sharma A. Kincl T. . (2025). Unveiling mechanism of SNSs addiction on wellbeing: the moderating role of loneliness and social anxiety. Behav. Inf. Technol. 44, 2876–2895. doi: 10.1080/0144929x.2024.2417390

[ref5] Ball-Rokeach S. J. DeFleur M. L. (1976). A dependency model of mass-media effects. Commun. Res. 3, 3–21. doi: 10.1177/009365027600300101

[ref6] Bronfenbrenner U. (2013). “Ecology of the family as a context for human development: Research perspectives,” in Adolescents and their families. Eds. R. M. Lerner and D. R. Castellino (New York, NY: Routledge).

[ref7] Baumeister R. F. (2002). Ego depletion and self-control failure: an energy model of the self's executive function. Self Identity 1, 129–136. doi: 10.1080/152988602317319302

[ref8] Busch P. A. McCarthy S. (2021). Antecedents and consequences of problematic smartphone use: a systematic literature review of an emerging research area. Comput. Hum. Behav. 114:106414. doi: 10.1016/j.chb.2020.106414

[ref9] Bond F. W. Hayes S. C. Baer R. A. Carpenter K. M. Guenole N. Orcutt H. K. . (2011). Preliminary psychometric properties of the acceptance and action questionnaire–II: a revised measure of psychological inflexibility and experiential avoidance. Behav. Ther. 42, 676–688. doi: 10.1016/j.beth.2011.03.007, 22035996

[ref10] Byrne B. M. (2001). Structural equation modeling with AMOS: Basic concepts, applications, and programming. 1st Edn. Mahwah, NJ: Lawrence Erlbaum Associates.

[ref11] Chapman A. L. Gratz K. L. Brown M. Z. (2006). Solving the puzzle of deliberate self-harm: the experiential avoidance model. Behav. Res. Ther. 44, 371–394. doi: 10.1016/j.brat.2005.03.005, 16446150

[ref12] Cao J. Ji Y. Zhu Z. (2013). Reliability and validity of the Chinese version of the acceptance and action questionnaire-II (AAQ-II) in assessing college students. Chin. J. Ment. Health 27, 873–877.

[ref13] Chen Y. Zhang W. Zhong N. Zhao M. (2024). Motivations behind problematic short video use: a three-level meta-analysis. Telemat. Inform. 93:102196. doi: 10.1016/j.tele.2024.102196

[ref14] Copeland W. E. Keen R. Tong G. Shanahan L. (2024). Negative life events and emotional symptoms from ages 2 to 30 years. JAMA Netw. Open 7:e2429448. doi: 10.1001/jamanetworkopen.2024.29448, 39207754 PMC11362870

[ref15] Cavour-Więcławek N. Rogowska A. M. (2024). Does self-reported trait mindfulness contribute to reducing perceived stress in women who practice yoga and are physically active? Behav. Sci. 14:772. doi: 10.3390/bs14090772, 39335987 PMC11429056

[ref16] Di Fabio A. Tsuda A. (2018). The psychology of harmony and harmonization: advancing the perspectives for the psychology of sustainability and sustainable development. Sustainability 10:4726. doi: 10.3390/su10124726

[ref17] Duradoni M. Serritella E. Avolio C. Arnetoli C. Guazzini A. (2022). Development and validation of the digital life balance (DLB) scale: a brand-new measure for both harmonic and disharmonic use of ICTs. Behav. Sci. 12:489. doi: 10.3390/bs12120489, 36546972 PMC9774106

[ref18] Duradoni M. Severino F. P. Bellotti M. Guazzini A. (2024a). How mattering and anti-mattering experiences across offline and online environments contribute to people's digital life balance and social media addiction. J. Community Appl. Soc. Psychol. 34:e70008. doi: 10.1002/casp.70008

[ref19] Duradoni M. Serritella E. Severino F. P. Guazzini A. (2024b). Exploring the relationships between digital life balance and internet social capital, loneliness, fear of missing out, and anxiety. Hum. Behav. Emerg. Technol. 2024:5079719. doi: 10.1155/2024/5079719

[ref20] Duradoni M. Colombini G. Barucci C. Zagaglia V. Guazzini A. (2025). Psychopathological correlates of dysfunctional smartphone and social media use: the role of personality disorders in technological addiction and digital life balance. Eur. J. Investig. Health Psychol. Educ. 15:136. doi: 10.3390/ejihpe15070136, 40709969 PMC12294652

[ref21] Everitt B. S. (1975). Multivariate analysis: the need for data, and other problems. Br. J. Psychiatry 126, 237–240. doi: 10.1192/bjp.126.3.237, 1125504

[ref22] Faul F. Erdfelder E. Buchner A. Lang A. G. (2009). Statistical power analyses using G*power 3.1: tests for correlation and regression analyses. Behav. Res. Methods 41, 1149–1160. doi: 10.3758/brm.41.4.1149, 19897823

[ref23] Feng T. Wang B. Mi M. Ren L. Wu L. Wang H. . (2025). The relationships between mental health and social media addiction, and between academic burnout and social media addiction among Chinese college students: a network analysis. Heliyon 11:e41869. doi: 10.1016/j.heliyon.2025.e41869, 39959490 PMC11830321

[ref24] Geng Y. Gu J. Yu J. Zhu X. (2020). Negative life events and depressive symptoms among Chinese adolescents: mediating role of resilience and moderating role of psychopathy. Curr. Psychol. 41, 1486–1493. doi: 10.1007/s12144-020-00621-7

[ref25] Garnefski N. Kraaij V. Spinhoven P. (2001). Negative life events, cognitive emotion regulation and emotional problems. Pers. Individ. Dif. 30, 1311–1327. doi: 10.1016/s0191-8869(00)00113-6

[ref26] Gámez W. Chmielewski M. Kotov R. Ruggero C. Watson D. (2011). Development of a measure of experiential avoidance: the multidimensional experiential avoidance questionnaire. Psychol. Assess. 23, 692–713. doi: 10.1037/a0023242, 21534697

[ref27] Gollust S. E. Eisenberg D. Golberstein E. (2008). Prevalence and correlates of self-injury among university students. J. Am. Coll. Heal. 56, 491–498. doi: 10.3200/jach.56.5.491-498 18400660

[ref28] Gong X. Xie X. Xu R. . (2010). Validation of the simplified Chinese version of the depression anxiety stress Scale-21 (DASS-21) in Chinese college students. Chin. J. Clin. Psychol. 18, 443–446.

[ref29] González-Fuente B. Moral-Jiménez M. D. L. V. (2024). Online and offline shopping addiction and its relationship with state-trait anxiety and impulsivity. Behav. Psychol. / Psicol. Conductual 32, 249–267. doi: 10.51668/bp.8324202n

[ref30] Gorsuch R. L. (2014). Factor analysis: classic edition. New York: Routledge.

[ref31] Holden C. (2000). Global survey examines impact of depression. Science 288, 39–40. doi: 10.1126/science.288.5463.39, 10766633

[ref32] Hayes S. C. (2004). Acceptance and commitment therapy, relational frame theory, and the third wave of behavioral and cognitive therapies. Behav. Ther. 35, 639–665. doi: 10.1016/s0005-7894(04)80013-3 27993338

[ref33] Hayes A. F. (2017). Introduction to mediation, moderation, and conditional process analysis: a regression-based approach. New York, NY: Guilford Publications.

[ref34] Hair J. Black W. Babin B. Anderson R. (2010). Multivariate data analysis: a global perspective. Upper Saddle River, NJ: Pearson Prentice Hall.

[ref35] He C. Wei H. Yan C. Wang C. (2021). The impact of cyberbullying on adolescent self-injury: the sequential mediation effect of depression and experiential avoidance. Chin. J. Clin. Psychol. 29, 338–342.

[ref36] Hu H. Huang M. (2024). How stress influences short video addiction in China: an extended compensatory internet use model. Front. Psychol. 15:1470111. doi: 10.3389/fpsyg.2024.1470111, 39583000 PMC11582829

[ref37] Kardefelt-Winther D. (2014). A conceptual and methodological critique of internet addiction research: towards a model of compensatory internet use. Comput. Hum. Behav. 31, 351–354. doi: 10.1016/j.chb.2013.10.059

[ref38] Kashdan T. B. Farmer A. S. Adams L. M. Ferssizidis P. McKnight P. E. Nezlek J. B. (2013). Distinguishing healthy adults from people with social anxiety disorder: evidence for the value of experiential avoidance and positive emotions in everyday social interactions. J. Abnorm. Psychol. 122, 645–655. doi: 10.1037/a0032733, 23815396

[ref39] Kuo A. Lutz R. J. Hiler J. L. (2016). Brave new world of Warcraft: a conceptual framework for active escapism. J. Consum. Mark. 33, 498–506. doi: 10.1108/jcm-04-2016-1775

[ref40] Kuo H. J. Yeomans M. Ruiz D. Lin C. C. (2024). Video games and disability: a risk and benefit analysis. Front. Rehabil. Sci. 5:1343057. doi: 10.3389/fresc.2024.1343057, 38496777 PMC10943698

[ref41] Lerner R. M. Lerner J. V. Almerigi J. . (2006). “Dynamics of individual-context relations in human development: a developmental systems perspective” in Comprehensive handbook of personality and psychopathology. Eds. M. Hersen and J. C. Thomas (Hoboken, NJ, USA: John Wiley & Sons Inc).

[ref42] Li D. Zhang W. Li X. Zhen S. Wang Y. (2010). Stressful life events and problematic internet use by adolescent females and males: a mediated moderation model. Comput. Hum. Behav. 26, 1199–1207. doi: 10.1016/j.chb.2010.03.031

[ref43] Li L. Li X. Li Y. Liu X. P. Huang L. (2024). Types of short video addiction among college freshmen: effects on career adaptability, insomnia, and depressive symptoms. Acta Psychol. 248:104380. doi: 10.1016/j.actpsy.2024.104380, 38955033

[ref44] Li S. Zhao T. Feng N. Chen R. Cui L. (2025). Why we cannot stop watching: tension and subjective anxious affect as central emotional predictors of short-form video addiction. Int. J. Ment. Health Addict., 23, 1–15. doi: 10.1007/s11469-025-01486-2

[ref45] Lima-Costa A. R. Tosti A. E. Bonfá-Araujo B. Duradoni M. (2024). Digital life balance and need for online social feedback: cross–cultural psychometric analysis in Brazil. Hum. Behav. Emerg. Technol. 2024:1179740. doi: 10.1155/2024/1179740

[ref46] Liao M. (2024). Analysis of the causes, psychological mechanisms, and coping strategies of short video addiction in China. Front. Psychol. 15:1391204. doi: 10.3389/fpsyg.2024.1391204, 39165759 PMC11333346

[ref47] Liu C. Chen H. Zhang A. Gong X. Wu K. Liu C. Y. . (2023). The effects of short video app-guided loving-kindness meditation on college students’ mindfulness, self-compassion, positive psychological capital, and suicide ideation. Psicol. Reflex. Crit. 36:32. doi: 10.1186/s41155-023-00276-w, 37902928 PMC10616025

[ref48] Laier C. Wegmann E. Brand M. (2018). Personality and cognition in gamers: avoidance expectancies mediate the relationship between maladaptive personality traits and symptoms of internet-gaming disorder. Front. Psych. 9:304. doi: 10.3389/fpsyt.2018.00304, 30042702 PMC6048288

[ref49] Longstreet P. Brooks S. Gonzalez E. S. (2019). Internet addiction: when the positive emotions are not so positive. Technol. Soc. 57, 76–85. doi: 10.1016/j.techsoc.2018.12.004

[ref50] Muraven M. Tice D. M. Baumeister R. F. (1998). Self-control as a limited resource: regulatory depletion patterns. J. Pers. Soc. Psychol. 74, 774–789, 9523419 10.1037//0022-3514.74.3.774

[ref51] Mehrabian A. Russell J. A. (1974). The basic emotional impact of environments. Percept. Mot. Skills 38, 283–301. doi: 10.2466/pms.1974.38.1.283, 4815507

[ref52] Malas O. Khan M. Zubair A. Guazzini A. Duradoni M. (2025). Psychometric validation of the digital life balance scale in Urdu and its relationship with life satisfaction, social media addiction, and internet addiction. Hum. Behav. Emerg. Technol. 2025:7873343. doi: 10.1155/hbe2/7873343

[ref53] Nong W. He Z. Ye J. H. Wu Y. F. Wu Y. T. Ye J. N. . (2023). The relationship between short video flow, addiction, serendipity, and achievement motivation among Chinese vocational school students in the post-epidemic era. Healthcare 11:462. doi: 10.3390/healthcare11040462 36832995 PMC9957412

[ref54] Nguyen T. T. Veer E. (2024). Why people watch user-generated videos? A systematic review and meta-analysis. Int. J. Hum.-Comput. Stud. 181:103144. doi: 10.1016/j.ijhcs.2023.103144

[ref55] Ozaki C. C. Olson A. B. Johnston-Guerrero M. P. Pizzolato J. E. (2020). Understanding persistence using a phenomenological variant of ecological systems theory. Community Coll. Rev. 48, 252–276. doi: 10.1177/0091552120906884

[ref56] Prievara D. K. Piko B. F. Luszczynska A. (2019). Problematic internet use, social needs, and social support among youth. Int. J. Ment. Health Addict. 17, 1008–1019. doi: 10.1007/s11469-018-9973-x

[ref57] Pu Y. Yang D. Yan W. (2023). The relationship between negative life events and short-video addiction among college students: the mediating role of self-compensatory motivation. J. Nanjing Univ. Chin. Med. Soc. Sci. Ed. 24, 204–210.

[ref58] Qin Y. Omar B. Musetti A. (2022). The addiction behavior of short-form video app TikTok: the information quality and system quality perspective. Front. Psychol. 13:932805. doi: 10.3389/fpsyg.2022.932805, 36148123 PMC9486470

[ref59] Qu D. Liu B. Jia L. Zhang X. Chen D. Zhang Q. . (2024). The longitudinal relationships between short video addiction and depressive symptoms: a cross-lagged panel network analysis. Comput. Human Behav. 152:108059. doi: 10.1016/j.chb.2023.108059

[ref60] Shao Y. J. Zheng T. Wang Y. Q. Liu L. Chen Y. Yao Y. S. (2018). Internet addiction detection rate among college students in the people’s republic of China: a meta-analysis. Child Adolesc. Psychiatry Ment. Health 12:25. doi: 10.1186/s13034-018-0231-6, 29849754 PMC5970523

[ref61] Sung G. Park Y. Choi T. K. Park S. W. (2020). Implicit theories and depression in clinical and non-clinical samples: the mediating role of experiential avoidance. Curr. Psychol. 39, 68–73. doi: 10.1007/s12144-017-9736-z

[ref62] Soysal F. S. O. Kosar E. Gursesli M. C. Guazzini A. Duradoni M. (2024). Digital life balance scale: validity and reliability in the Turkish context. Hum. Behav. Emerg. Technol. 2024, 1–9. doi: 10.1155/2024/9454784, 41293084

[ref63] Tosti A. E. Tereshchenko S. Evert L. Guazzini A. Duradoni M. (2025). The digital life balance scale: validation and gender invariance among urban Russian adolescents. Int. J. Hum.-Comput. Interact. 41, 1–12. doi: 10.1080/10447318.2025.2520934

[ref64] Updegraff J. A. Taylor S. E. (2021). “From vulnerability to growth: positive and negative effects of stressful life events” in Loss and trauma. Eds. J. H. Harvey and E. Miller (Milton Park: Routledge).

[ref65] Wei H. Li Y. Liu M. He A. (2023). Parental phubbing and adolescent suicidal ideation: a perspective from the experiential avoidance model. Chin. J. Clin. Psychol. 31, 246–249.

[ref66] Xue J. Huang H. Guo Z. Chen J. Feng W. (2025). Adverse childhood experiences and short-form video addiction: a serial mediation model of resilience and life satisfaction. Comput. Hum. Behav. 162:108449. doi: 10.1016/j.chb.2024.108449, 41314920

[ref67] Yang Z. (2023). Why adolescents are addicted to social media. J. Educ. Humanit. Soc. Sci. 8, 1430–1436. doi: 10.54097/ehss.v8i.449

[ref68] Yao N. Chen J. Huang S. Montag C. Elhai J. D. (2023). Depression and social anxiety in relation to problematic TikTok use severity: the mediating role of boredom proneness and distress intolerance. Comput. Hum. Behav. 145:107751. doi: 10.1016/j.chb.2023.107751

[ref69] Yosep I. Suryani S. Mediani H. S. Mardhiyah A. Ibrahim K. (2024). Types of digital mindfulness: improving mental health among college students–a scoping review. J. Multidiscip. Healthc., 17, 43–53. doi: 10.2147/jmdh.s44378 38205126 PMC10777865

[ref70] Ye J. H. Wu Y. T. Wu Y. F. Chen M. Y. Ye J. N. (2022). Effects of short video addiction on the motivation and well-being of Chinese vocational college students. Front. Public Health 10:847672. doi: 10.3389/fpubh.2022.847672, 35619803 PMC9127725

[ref71] Ye J. H. Wu Y. F. Nong W. Wu Y. T. Ye J. N. Sun Y. (2023a). The association of short-video problematic use, learning engagement, and perceived learning ineffectiveness among Chinese vocational students. Healthcare 11:161. doi: 10.3390/healthcare11020161, 36673529 PMC9858663

[ref72] Ye J. H. He Z. Yang X. Lee Y. S. Nong W. Ye J. N. . (2023b). Predicting the learning avoidance motivation, learning commitment, and silent classroom behavior of Chinese vocational college students caused by short video addiction. Healthcare 11:985. doi: 10.3390/healthcare11070985, 37046912 PMC10094292

[ref73] Ye J. H. Cui Y. Wang L. Ye J. N. (2024a). The relationships between short video addiction, self-regulated learning, and learning well-being of Chinese undergraduate students. Int. J. Ment. Health Promot. 26, 805–815. doi: 10.32604/ijmhp.2024.055814

[ref74] Ye J. H. Chen M. Y. Wu Y. F. (2024b). The causes, counseling, and prevention strategies for maladaptive and deviant behaviors in schools. Behav. Sci. 14:118. doi: 10.3390/bs14020118, 38392471 PMC10885922

[ref75] Ye J. H. Zheng J. Nong W. Yang X. (2025). Potential effect of short video usage intensity on short video addiction, perceived mood enhancement (“TikTok brain”), and attention control among Chinese adolescents. Int. J. Ment. Health Promot. 27, 271–286. doi: 10.32604/ijmhp.2025.059929

[ref76] Yu Z. Zhu X. Li Y. (2024). The association between problematic short video use and suicidal ideation and self-injurious behaviors: the mediating roles of sleep disturbance and depression. BMC Public Health 24:1689. doi: 10.1186/s12889-024-19191-5, 38915039 PMC11197212

[ref77] Zahra M. F. Qazi T. A. Ali A. S. Hayat N. ul Hassan T. (2022). How TikTok addiction leads to mental health illness? Examining the mediating role of academic performance using structural equation modeling. J. Posit. Sch. Psychol. 6, 1490–1502. doi: 10.1007/s11469-021-00541-y

[ref78] Zhang M. Bian Y. (2021). An analysis of the brain structures underlying the link between pathological internet use and anxiety. Addict. Behav. 112:106632. doi: 10.1016/j.addbeh.2020.106632, 32905867

[ref79] Zhang L. Zhuo X. F. Xing K. Liu Y. Lu F. Zhang J. Y. . (2024). The relationship between personality and short video addiction among college students is mediated by depression and anxiety. Front. Psychol. 15:1465109. doi: 10.3389/fpsyg.2024.1465109, 39534468 PMC11555565

[ref80] Zhang Z. Y. Wu Y. Deng C. Wang P. Nong W. (2025). Relationship between Chinese medical students’ perceived stress and short-form video addiction: a perspective based on the multiple theoretical frameworks. Int. J. Ment. Health Promot. 27, 1533–1551. doi: 10.32604/ijmhp.2025.070883

[ref81] Zhou Z. Hu X. Zhang Y. Su R. Xin S. Sheng J. (2025). The relationship between negative life events and internet addiction among middle school students: the roles of social anxiety and boredom proneness. Psychol. Dev. Educ. 41, 109–116.

